# Research on the Synthesis and Conductivity of Titanium Oxycarbide

**DOI:** 10.3390/ma18194621

**Published:** 2025-10-06

**Authors:** Shaolong Li, Fan Yang, Peizhu Mao, Tianzhu Mu, Fuxing Zhu, Shengwei Li

**Affiliations:** 1 State Key Laboratory of Vanadium and Titanium Resources Comprehensive Utilization, Pangang Group Research Institute Co., Ltd., Panzhihua 617000, China; 15205199180@163.com (T.M.); he150f@163.com (F.Z.); 2 School of Material Science and Engineering, Zhengzhou University, Science Road 100, Zhengzhou 450001, China; dbchcy178@gs.zzu.edu.cn (F.Y.); 13237641651@163.com (P.M.); 3 National Key Laboratory of Special Rare Metal Materials, Zhengzhou University, Science Road 100, Zhengzhou 450001, China; 4 Western Mining Group Co., Ltd., Xining 810000, China

**Keywords:** carbothermal reduction method, TiC*_x_*O*_y_*, C/O molar ratio

## Abstract

In this study, TiC*_x_*O*_y_* was produced by sintering in an argon atmosphere using carbon–thermal reduction with TiO_2_ and graphite powder as the initial materials. The sintered TiC*_x_*O*_y_* was analyzed using X-ray diffraction, scanning electron microscopy, and energy-dispersive X-ray spectroscopy. As the oxygen content increased, the grain color of the sintered TiC*_x_*O*_y_* gradually shifted from gray to reddish-brown. The structure of TiC*_x_*O*_y_* resembles that of a coral, with a uniform distribution of Ti, C, and O throughout the sample. Analysis using X-ray photoelectron spectroscopy reveals the presence of bivalent, trivalent, and tetravalent titanium. Utilizing General Structure Analysis System software (GSAS-II), the X-ray Diffraction data obtained were refined, revealing a gradual decrease in lattice parameters as the oxygen atom content increased. Furthermore, the conductivity and density of the single phase, determined through the four-probe method and the Archimedes method, respectively, exhibited an increase in tandem with the rise in C content.

## 1. Introduction

Titanium oxycarbide is classified as a solid solution, wherein oxygen and carbon are situated in octahedral interstitial positions within the face-centered cubic structure of titanium [1,2,3,4,5]. When it is assumed to be an ideal crystal, the compound is usually represented by the formula TiC*_x_*O_1−*x*_. In the early phases of study, TiC*_x_*O*_y_* was mainly explored as a thin film [6,7,8]. Presently, studies concerning titanium oxycarbide predominantly concentrate on its distinctive features and the potential applications across different sectors. Titanium oxycarbide is recognized for its outstanding electrical and thermal conductivity, formidable mechanical properties, reliable structural stability, extraordinary hardness, and high melting point. Consequently, it has been extensively utilized in the area of surface decoration [9,10,11]. Moreover, titanium oxycarbide is recognized as a proficient catalytic carrier material, commonly applied as an electron conduction carrier for electrochemical energy storage applications.

At present, the primary methods for producing TiC_x_O_y_ materials are as follows: DC magnetron sputtering via using C pieces incrusted in a Ti target erosion area [12]; electrochemical synthesis, which refers to the method of producing titanium oxycarbide by utilizing TiO_2_ and carbon in a molten salt medium [5,13]—this approach is comparable to the Fray–Farthing–Chen (FFC) method [14]; vacuum heat treatment of TiO_2_ and TiC [15]; high-temperature gas–solid combustion method [16]; and another commonly utilized method for producing titanium oxycarbide is the carbothermal reduction process [17]. This method is conducted in an argon atmosphere, employing titanium dioxide as the raw material and carbon as the reducing agent. The fundamental nature of this process is a solid-phase reaction that involves the participation of gases.

The synthesis of TiC_x_O_y_ through carbothermal reduction in an argon atmosphere has been extensively researched [17]; however, the characteristics of the resulting TiC_x_O_y_ exhibit significant variability. This variation is primarily attributed to the differing carbon–oxygen ratios, which influence the crystal structure, electronic properties, and chemical stability of the material. Research conducted in the past has revealed that a greater carbon content, denoted by an increased C/O ratio, generally results in improved conductivity of materials [18]. This improvement manifests as a transition from semiconductor behavior to metallic behavior in conductivity. The reason for this transition is attributed to the fact that carbon can introduce a larger number of free electrons, which enhances charge transport capabilities. When the C/O ratio is lower, it signifies a higher concentration of oxygen, potentially increasing the material’s vulnerability to oxidation and resulting in lower chemical stability. On the other hand, a higher C/O ratio contributes to improved oxidation resistance, thereby providing greater stability in oxidizing conditions [8]. Furthermore, variations in the carbon–oxygen ratio will influence the material’s susceptibility to corrosion in aggressive environments, including acids and alkalis. Generally, an increased C/O ratio tends to improve the corrosion resistance of the material [8]. It has been observed that an increased C/O ratio leads to a material with greater hardness. Nevertheless, some reports indicate that this relationship does not follow a linear pattern. Notably, when the C/O ratio reaches 3:1, the hardness of the material approaches that of TiC [7]. TiC_x_O_y_ is also applicable as a consumable anode material in electrolysis; however, a C/O ratio greater than 1 will lead to the emergence of anode C mud during the consumption of the anode. The existence of this anode mud is not beneficial for the metal smelting process. It has been reported that for TiC_x_O_y_ to function effectively as a depleting anode, the ratio of oxygen to carbon must be maintained at approximately one, or C/O≈1, in order to mitigate the influences of both carbon and oxygen [19]. Simultaneously, Fernandes et al. [20] put forth the idea that TiC_x_O_y_ materials demonstrate significant hardness and Young’s modulus when the C/O ratio is equal to 1. Additionally, the distinct C/O ratios affect the catalytic and optical attributes of the material [21]. Consequently, accurate regulation of the C/O ratio in titanium oxycarbide is vital for optimizing its functionality to align with specific application demands.

Although numerous studies have explored the synthesis of TiC*_x_*O*_y_* powders or thin films, our work systematically investigates the combined effects of precursor ratio (C/O) and sintering temperature on the formation of single-phase TiC*_x_*O*_y_* bulk ceramics via carbothermal reduction—an aspect less explored in prior studies. Furthermore, unlike many existing reports that focus solely on structural characterization, our research provides a comprehensive correlation among composition, lattice parameter, microstructure, electrical conductivity, and density. This integrated approach offers new insights into the controllability of functional properties in TiC*_x_*O*_y_* systems, which is critical for their potential applications in conductive ceramics and functional coatings.

## 2. Materials and Methods

**Figure 1:**
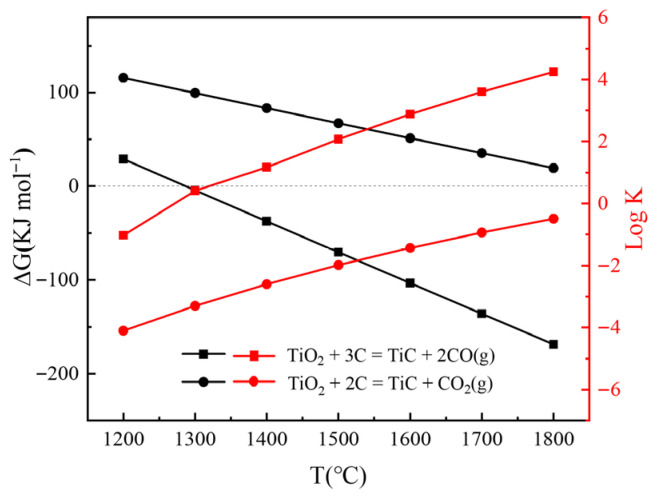
Gibbs free energy change and reaction equilibrium constant of TiO_2_ reacting with C in argon atmosphere (data from HSC 6.0).

Figure 1 shows the relationship between temperature and Gibbs free energy (ΔG) and equilibrium constant (log K) of TiO_2_ reaction with C by HSC 6.0 software in argon atmosphere.

TiO_2_ + 3C = TiC + 2CO(g) (1)

TiO_2_ + 2C = TiC + CO_2_(g) (2)

TiO_2_ + C → TiC*_x_*O*_y_* + CO(g) (3)

It is evident that the ΔG values of Equations (1) and (2) decrease as the temperature rises. This indicates that they are endothermic reactions, and higher temperatures are conducive to the occurrence of these reactions. In Equation (1), C reacts with TiO_2_ to form TiC and CO when the temperature exceeds 1300 °C. In Equation (2), when the temperature reaches 1800 °C, the Δ G value of the reaction remains positive, indicating that TiO_2_ will not react with C to form TiC and CO_2_. Therefore, in the carbothermal reduction process of TiO_2_, the final gas product is CO, not CO_2_. When TiC*_x_*O*_y_* is generated, the reaction proceeds according to Equation (3). In this study, the synthesis temperature was set to 1200~1600 °C. The molar ratio of raw materials TiO_2_ to graphite was 1:2.0, the atmosphere was argon, and the dwell time was 4 h, investigating the influence of temperature on the phase composition and microstructure of the product. Heating at 1600 °C was ultimately selected as the optimum temperature, the holding time was 4 h, and the holding atmosphere was argon. By adjusting the ratio of TiO_2_ and graphite in raw materials, the phase and microstructure changes in the products were investigated.

**Figure 2:**
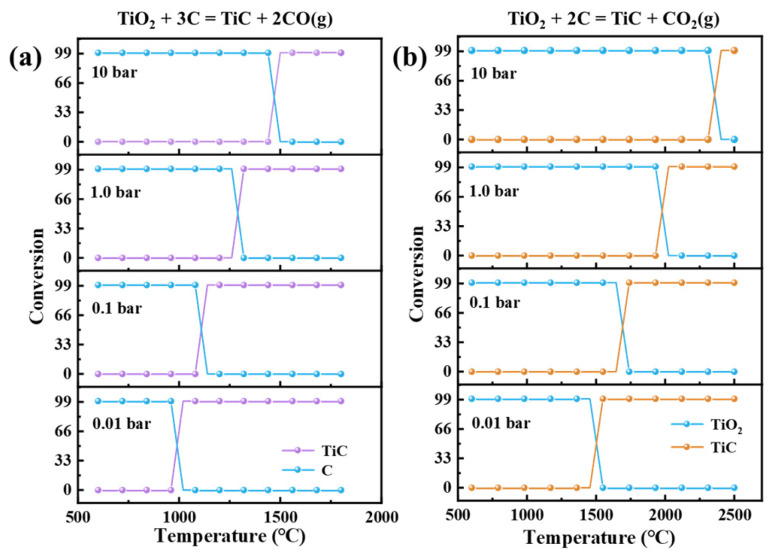
The relationship between Equilibrium compositions and temperature under different partial pressures. (data from HSC 6.0). (**a**) reactions (1); (**b**) reactions (2).

Figure 2 displays the equilibrium composition distribution of Equations (1) and (2) under different partial pressures. As the gas-phase pressure increases, the reactions become less favorable. Figure 2b demonstrates that the equilibrium temperature for Equation (2) must significantly exceed 1600 °C. Consequently, this reaction cannot proceed at 1600 °C in an argon atmosphere, which aligns with the results shown in Figure 1.

The carbothermal reduction route was employed to produce TiC*_x_*O*_y_* powders from TiO_2_ (99.99 pct pure, Aladdin, Shanghai, China) and graphite (>99.85 pct pure, ≤30 μm, Aladdin, Shanghai, China). Assuming TiC*_x_*O*_y_* has an ideal crystal structure without defects such as oxygen vacancies, the corresponding reaction can be expressed as Equation (4).

TiO_2_ + (1 + 2x) C = TiC*_x_*O*_1−x_* + (1 + x) CO(g) (4)

**Figure 3:**
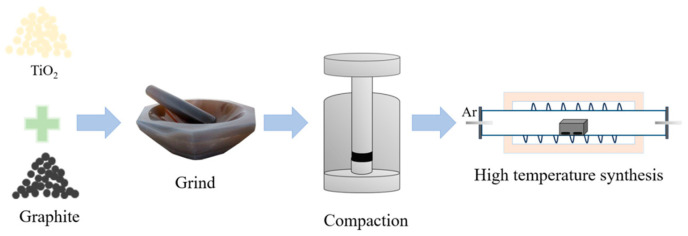
Schematic diagram of the synthesis process of TiC*_x_*O*_y_*.

In order to obtain TiC*_x_*O*_y_* of TiC_0_._7_O_0_._3_, TiC_0_._6_O_0_._4_, TiC_0.5_O_0.5_, TiC_0.4_O_0.6_ and TiC_0.3_O_0.7_ with different C to O ratios, according to Equation (4), The theoretical molar ratios of TiO_2_ and graphite should be 1:2.4, 1:2.2, 1:2.0, 1:1.8 and 1:1.6, respectively. The synthesis process of TiC*_x_*O*_y_* is shown in Figure 3.

The raw materials are weighed in proportion and then ground in a mortar to obtain a uniform mixture of evenly mixed TiO_2_ and graphite powder. In general, compaction can increase the effective contact area between the mixture, thereby speeding up the reaction process and shortening the reaction time. Therefore, in this experiment, we used an electric tablet press (DYPD-30T) to press the above evenly mixed powder. The mold material used was stainless steel, the shape of the press block was a round cake, the diameter was 12.5 mm, the mass of each sample was 1.2 ± 0.05 g, and the pressure was set to 100 MPa. The pressed sample is placed in a graphite crucible with a graphite lid and then sintered in a tubular sintering furnace. The sintering process was carried out at 1600 °C in high purity argon atmosphere (flow rate was 25 mL/min), and the holding time was 4 h. Cool to room temperature to obtain the target product.

Utilizing X-ray Diffraction (XRD-6100, SHIMADZU, Duisburg, Germany, 0.02° step size, Cu Kα radiation), the phase purity of the sintered sample was assessed. Scanning Electron Microscopy (SEM, TESCAN MIRA LMS, Brno, Czech Republic and ZEISS Sigma 300, Dortmund, Germany) was employed to investigate the microscopic morphology and elemental distribution of the synthesized samples under various parameters, and the elemental content in the electrolytic products was qualitatively analyzed by Energy Dispersive Spectrometer (EDS, Berlin, Germany). The XRD patterns obtained were refined using General Structure Analysis System software (GSAS II) with the Rietveld method to determine precise lattice parameters. Due to the similar X-ray scattering powers of carbon and oxygen, their site occupancies were constrained based on the nominal composition derived from the starting mixtures. Resistivity measurements of the sintered TiCO samples were conducted using a four-point probe technique (RTS-8, Jandel Engineering, Leighton Buzzard, UK). The system consisted of a Signatone S-302-4 probe station equipped with a four-point head and a Keithley 2450 Source Measure Unit (Tektronix, Beaverton, OR, USA). To minimize edge effects, samples with dimensions exceeding 10 times the probe spacing were used. All measurements were performed at room temperature (25 ± 0.5 °C) in a shielded environment to minimize electromagnetic interference.

## 3. Results

**Figure 4:**
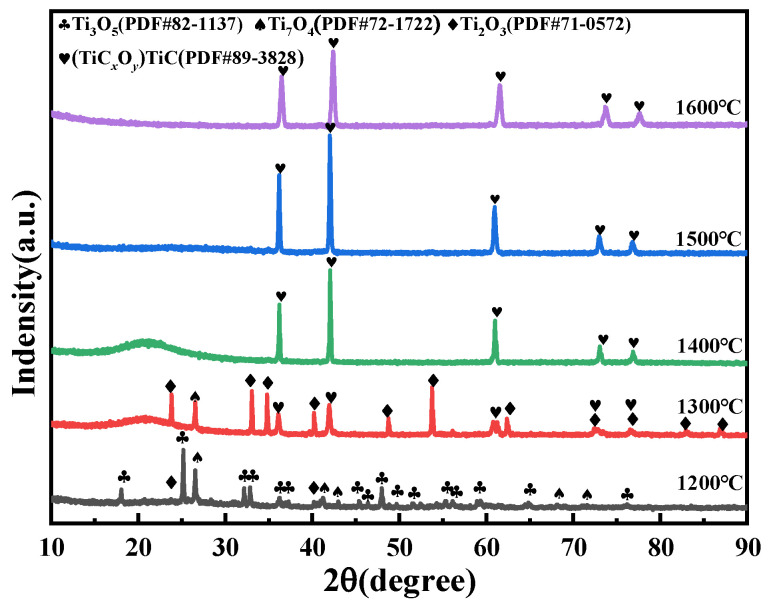
XRD patterns and optical photographs of samples after holding TiO_2_ and graphite (molar ratio of 1:2) in argon atmosphere at different temperatures for 4 h.

To investigate the effect of temperature on the sintered products, samples with a molar ratio of TiO_2_ to graphite of 1:2.0 were sintered at different temperatures, all held for 4 h. The XRD patterns of the resulting products are shown in Figure 4. The results indicate that the reduction begins at ~1200 °C with the formation of lower titanium oxides (e.g., Ti_3_O_5_) through a solid-state reaction. The formation of titanium oxycarbide (TiC*_x_*O*_y_*) is a critical intermediate step, and its composition evolves as oxygen is progressively replaced by carbon. The reduction sequence will be described as a progressive process: TiO_2_ → Ti*_n_*O_2*n*−1_ (Magnéli phases) → TiO → TiC*_x_*O*_y_*. We will compare our findings with established mechanisms from key literature sources [22]. This will demonstrate that our experimental results are consistent with the known stepwise reduction mechanism.

**Figure 5:**
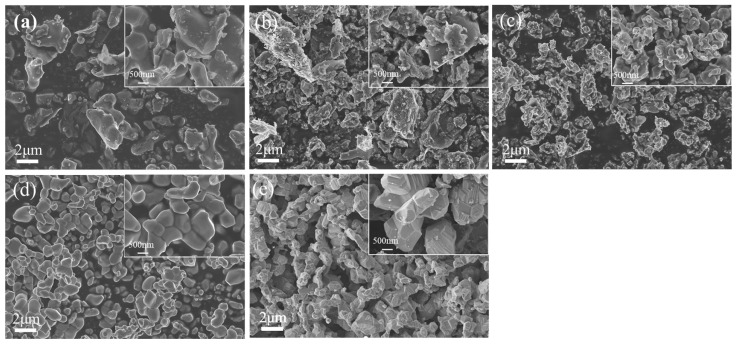
Micro-morphology of samples after holding TiO_2_ and graphite (molar ratio of 1:2) in argon atmosphere at different temperatures for 4 h. (**a**) 1200 °C; (**b**) 1300 °C; (**c**) 1400 °C; (**d**) 1500 °C; (**e**) 1600 °C.

Figure 5 displays the microstructures of the samples after heating at different temperatures. Following heating at 1200 °C, TiO_2_ particles within the sample were observed to agglomerate, and flaky graphite remained distinctly visible. As the temperature increased to 1300 °C and 1400 °C, the sample surfaces exhibited relatively smooth edges. A significant change in microstructure occurred at 1500 °C: flaky graphite was almost entirely absent, and a new phase emerged. At 1600 °C, the presence of the new phase became more prominent and the surface edges developed a terraced morphology. These terraced features represent traces left by the reaction between TiO_2_ and graphite, indicating that the reaction progressed more extensively at elevated temperatures. The observed evolution in microstructure aligns with the phase transformation trends identified by XRD. The enhanced progression of the reaction with increasing temperature led to the selection of 1600 °C as the optimal reaction temperature in this experiment.

**Figure 6:**
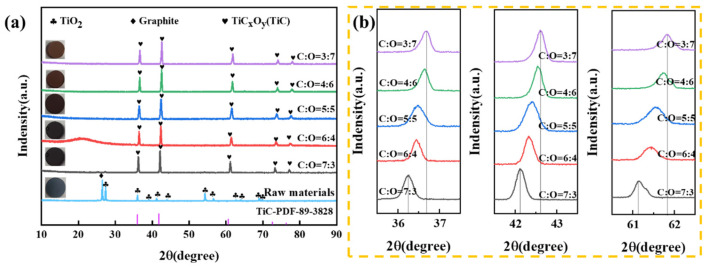
(**a**) XRD patterns of TiO_2_ and graphite mixtures with different molar ratios held at 1600 °C in argon atmosphere for 4 h; (**b**) Diffraction peak displacement of TiC*_x_*O*_y_* phase.

The sample phase can be observed in Figure 6a. Prior to the reaction, the XRD pattern of the raw material exhibited the presence of graphite and TiO_2_ phases. Following sintering at a temperature of 1600 °C for a duration of 4 h, a notable phase transformation occurred. A novel diffraction peak emerged in the XRD analysis when compared to the diffraction peak of TiO_2_. Furthermore, the diffraction peak exhibited a significant shift towards a higher angle in comparison to TiC. According to the pertinent literature [23], it becomes evident that the reaction between TiO_2_ and graphite results in the formation of a single-phase compound known as TiC*_x_*O*_y_*. The phase of TiO_2_ and graphite was experimentally studied when the molar ratios of TiO_2_ and graphite were 1:2.4, 1:2.2, 1:2.0, 1:1.8 and 1:1.6, respectively (The carbon-to-oxygen ratios are 7:3, 6:4, 5:5, 4:6 and 3:7, respectively). Furthermore, there was a notable alteration in the color of the sintered sample, transitioning from gray to reddish-brown as the C content diminished [18]. Figure 6b shows a close-up view of the shift in diffraction peaks of TiC*_x_*O*_y_* phase. With the reduction of carbon content in the raw material, the proportion of carbon atoms in TiC*_x_*O*_y_* obtained after the reaction also decreases, the lattice parameters become smaller, and the corresponding diffraction peak shifts to a higher angle [24]. When the ratio of TiO_2_ and graphite in the raw material is reduced from 1:1.8 to 1:1.6, the angle deviation of diffraction peak is very small, indicating that the carbon content in the obtained TiC*_x_*O*_y_* remains relatively consistent.

**Figure 7:**
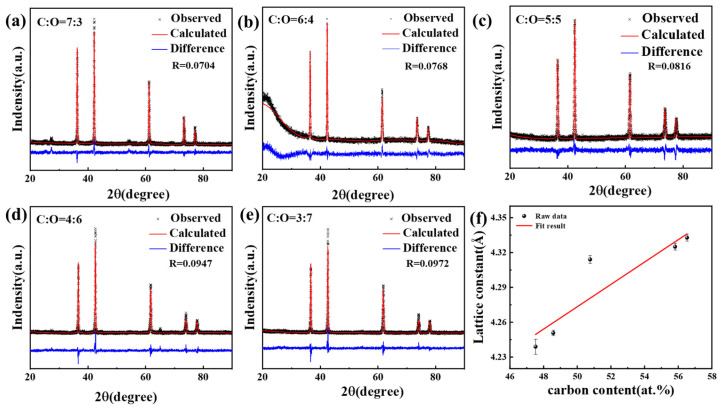
(**a**–**e**) The finishing curves and lattice parameters of TiC*_x_*O*_y_* (Ti/C molar ratios: 1:2.4, 1:2.2, 1:2.0, 1:1.8 and 1:1.6); (**f**) Analysis plot of lattice parameter versus carbon content.

Utilizing the GSAS-II software, the structure of the TiC*_x_*O*_y_* crystal shown was refined to determine the crystal structure and lattice constant of the TiC*_x_*O*_y_* solid solution [25] (Figure 7). The results indicate that the calculated diffraction peak is in good agreement with the measured diffraction peak, and the cell parameters are obtained from the least square results of the powder data. The difference between the experimental spectral line and the theoretical spectral line is small, and there is basically no residual diffraction peak, indicating that the structure is reasonable. However, a distinct peak observed at approximately 27° in Figure 7a is attributed to the presence of residual graphite or graphitic carbon phases that were not completely consumed during the sintering process. The specific Ti/C molar ratios are 1:2.4, 1:2.2, 1:2.0, 1:1.8 and 1:1.6 and the corresponding lattice constants are 4.33268 Å, 4.32528 Å, 4.31335 Å, 4.24997 Å and 4.24131 Å, respectively. According to the Bragg equation, the diffraction summit of the phase shifts to a higher angle as the lattice parameters decrease. The outcome is consistent with the results in Figure 6b.

**Figure 8:**
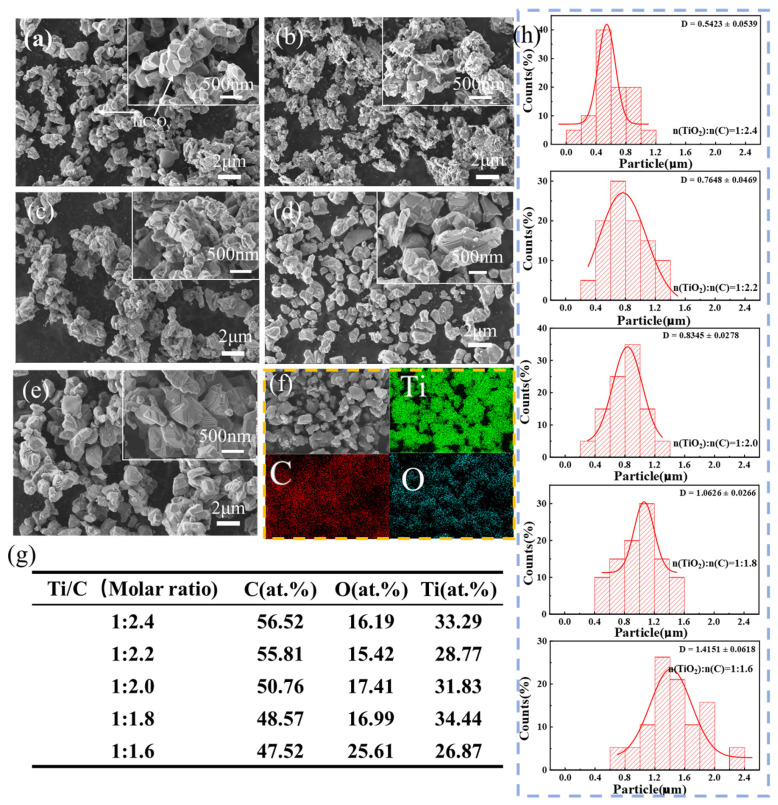
Microstructure and composition test results of TiO_2_ and graphite mixtures with different molar ratios after being held at 1600 °C in argon atmosphere for 4 h. (**a**) 1:2.4 (**b**) 1:2.2 (**c**) 1:2.0 (**d**) 1:1.8 (**e**) 1:1.6 (**f**) Elemental distribution diagram of the product when the raw material ratio was 1:2.0 (**g**) EDS component detection results in each region (**h**) Size distribution of TiC*_x_*O*_y_.*

Figure 8a–e shows the micrographs of the samples at different ratios of TiO_2_ and graphite powder after being held at 1600 °C for 4 h. It is possible to observe that the sample contains man*y* micrometer-sized grains that join together to form a coral-like structure with many holes. Upon closer inspection of the coral-like structure shown in Figure 6, it can be seen that the grain surface has a layered microstructure resembling a step, which indicates that the formation mechanism of TiC*_x_*O*_y_* is layer-by-layer growth [23]. The enlarged images in Figure 8a–e show a gradual decrease in nanoscale, which is due to the gradual reduction of carbon content in the sample and the reduction in the amount of substituted oxygen during the reaction with TiO_2_, resulting in a high oxygen content in the sample, so the grain size is gradually reduced. EDS was utilized to select random points from the aforementioned samples for point scanning to examine the element content, and qualitative analysis was performed on the samples according to the changes in element content. The analysis in Figure 8g indicates that the elemental composition is only composed of C, Ti, and O. With the decrease in C in the raw materials, the O content in the product shows an overall upward trend. Furthermore, we calculated the particle size distribution (Figure 8h), and the results were consistent with the aforementioned findings. To verify the composition of the synthesized material, the product was analyzed using carbon–sulfur and oxygen–nitrogen–hydrogen analyzers. The results indicated that the carbon (C) and oxygen (O) contents were determined to be 13.32 wt.% and 4.91 wt.%, respectively.

**Figure 9:**
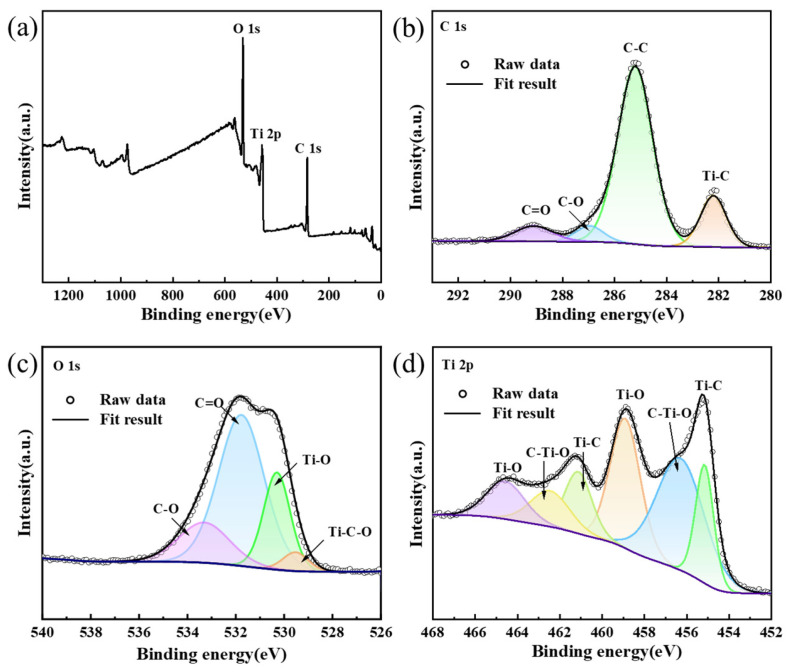
XPS spectra of TiC_0.5_O_0.5_. (**a**) Broad spectrum; (**b**) The C 1s region; (**c**) O 1s region; (**d**) Ti 2p region.

X-ray photoelectron spectroscopy (XPS) was performed on the prepared TiC_0.5_O_0.5_ sample to analyze the existence of titanium and its interactions with carbon and oxygen, with the objective of enhancing comprehension of the titanium valence state in TiC*_x_*O*_y_*. The obtained results are presented in Figure 9. The measurement spectrum shown in Figure 9a shows the presence of Ti, C, and O elements in the sample, which is consistent with the EDS analysis results shown in Figure 8. The binding energies of all elements were calibrated by C1s (284.8 eV), and the binding forms of each element could be expressed in more detail by fitting the narrow spectrum. According to reports in relevant literature [26], the highest peak of the four peaks in the C1s region corresponds to the C-C bond [27], the peak at 282.18 eV corresponds to the C-Ti bond, and the peak at 286.98 eV and 289.18 eV corresponds to the C-O and C=O bond respectively [28]. The existence of C-Ti bonds and C-O bonds suggests the formation of composite TiC*_x_*O*_y_* solid solutions. Since the binding energy of metal oxides is about 530 eV, and that of metal carbon oxides is about 528 eV [29,30], in the O 1s region shown in (c), the peak at 530.28 eV corresponds to the Ti-O bond, and the peak at 529.48 eV corresponds to the Ti-C-O bond. The remaining two peaks correspond to the C-O and C=O bonds, respectively. The spectrum shown in (d) is the XPS spectrum of Ti 2p, and the three main characteristic peaks corresponding to Ti-O, C-Ti-O and Ti-C respectively [29,31,32]. The existence of Ti-C and Ti-O bonds indicates that the TiC*_x_*O*_y_* solid solution within the composite structure leads to various bonding configurations of titanium.

**Figure 10:**
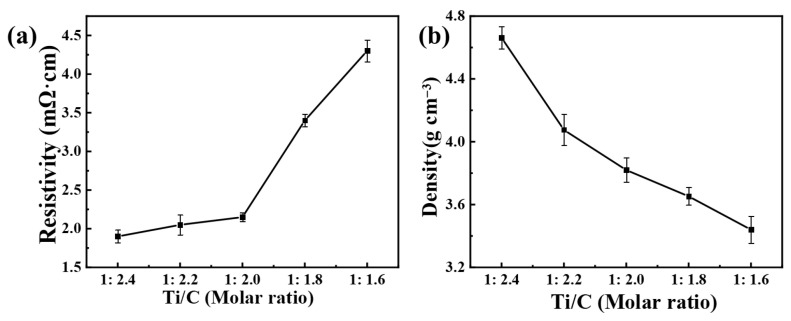
(**a**) relation resistivity of TiC*_x_*O*_y_*; (**b**) relation density of TiC*_x_*O*_y_*.

The conductivity of the TiC*_x_*O*_y_* solid solution was analyzed at different C/O ratios utilizing the four-probe method, and the density was measured via the Archimedes technique. Figure 10 presents the resistivity and density of TiC*_x_*O*_y_* solid solutions for various C/O ratios. As evident from the graphical data, the electrical resistivity of TiC*_x_*O*_y_* solid solutions exhibits a positive correlation with oxygen content, while their density demonstrates an inverse dependency under identical compositional variations [9]. The increase in carbon content in the product results in the weakening of the ionic bond between titanium and oxygen, while strengthening the covalent bond between titanium and carbon. In comparison with TiCO samples reported in the literature, our sample (TiC_0.5_O_0.5_) has a lower resistivity (3.4 mΩ·cm) and higher conductivity (294.23 s/cm) than Long et al. [33]. This may be attributed to we achieved lower oxygen content (<50 ppm vs. their 200 ppm) via vacuum sintering, minimizing charge carrier trapping. Furthermore, Our conductivity was measured at 300 K, where thermal activation enhances carrier mobility. We used Archimedes’ method (low surface tension) to minimize errors from open pores. The detailed test findings are presented in Table 1. In addition, we have included the corresponding properties of TiC*_x_*O*_y_* table, as shown in Table 2.

## 4. Conclusions

Within this study, TiO_2_ and graphite were utilized as raw materials to fabricate single-phase TiC*_x_*O*_y_* samples at high temperatures via carbothermal reduction method under an argon atmosphere. An in-depth examination was carried out to explore how the raw material ratio affects the microstructure, structure, and conductivity of sintered products. The following conclusions can be drawn from the above experimental results.

When the ratio of TiO_2_ to C is maintained at 1:2.4, 1:2.2, 1:2.0, 1:1.8, or 1:1.6, single-phase TiC*_x_*O*_y_* can be produced by holding the mixture at 1600 °C for a duration of 4 h in an argon atmosphere. The topography structure presents a coral-like. The XRD data acquired was refined using General Structure Analysis System software (GSAS-II). It was observed that as the oxygen atom content increased, the lattice parameters exhibited a gradual decrease. Subsequently, the conductivity and density of the resulting single phase were measured, revealing the following results: the conductivity of the TiC*_x_*O*_y_* solid solution increases as the oxygen content is reduced, whereas the density shows a decreasing trend with lower oxygen levels.

## Figures and Tables

**Table 1:** Resistivity and conductivity data measurements and averages.

Sample	Mean Resistivity (mΩ·cm)	Measurement Errors	Mean Conductivity (s/cm)
1:2.4	1.90	0.0817	527.05
1:2.2	2.05	0.1291	489.25
1:2.0	2.15	0.0577	459.93
1:1.8	3.40	0.0816	294.23
1:1.6	4.30	0.1414	232.78

**Table 2:** The properties of TiC*_x_*O*_y_.*

Category	Reference [22]	Reference [33]	This Work
Synthesis Method	Carbothermal reduction, 1550 °C, Ar	Ar + O_2_	Carbothermal reduction, 1600 °C, Ar
Composition	TiC_0.5_O_0.5_	TiC*_x_*O*_y_*	TiC_0.5_O_0.5_
Density	--	--	3.802 g/cm^3^
Resistivity	5.04 mΩ·cm	with increasing oxygen content	2.15 mΩ·cm

## Data Availability

The original contributions presented in the study are included in the article, further inquiries can be directed to the corresponding author.
